# Highlight: Taking a Closer Look at Experimental Evolution

**DOI:** 10.1093/gbe/evac124

**Published:** 2022-09-09

**Authors:** Casey McGrath

Humans have unwittingly been carrying out evolution experiments for millennia through the domestication of plants, animals, and fungi. Starting with the seminal experiments of William Dallinger in the late 19th century, such experiments have been performed under controlled laboratory conditions to better understand the processes and constraints of evolution. Evolutionary experiments generally involve imposing a well-defined selective pressure (such as extreme temperature, limited nutrients, or the presence of a toxic compound) on an organism and then studying how it adapts to these new conditions. The longest running controlled evolution experiment was begun in 1998 by Richard Lenski and continues to this day, involving over 60,000 generations of the bacterium *Escherichia coli*. While these experiments have provided foundational insight into evolutionary processes such as adaptation, selection, and mutation, it is clear that natural evolution occurs under much more complex constraints. A new study published in *Genome Biology and Evolution* sheds new light on the manner in which laboratory evolution may differ from what occurs in nature (Cohen and Hershberg 2022). According to co-author Ruth Hershberg, associate professor at Technion-Israel Institute of Technology, “Our results show that lab adaptation, which occurs in response to fairly simple and strong pressures, may often occur through mutations that either cannot occur in nature, or are very transient, if they do occur.”

The study, which was co-authored by Technion PhD student Yasmin Cohen, sought to explain an apparent paradox noticed by the authors when reflecting on the mutations identified in their own evolution experiments involving bacteria: namely, that the proteins in which mutations most often occur in the laboratory are the same as those that change most slowly over long evolutionary timescales. To further explore this observation, Cohen and Hershberg specifically looked at two genes encoding the RNA polymerase core enzyme (RNAPC), which were shown to be involved in adaptation within many independent laboratory evolution experiments in *E. coli*, the species most commonly used for these types of experiments. Their literature survey identified adaptive mutations at 140 amino acid positions across these proteins in response to 12 different laboratory conditions, including exposure to antibiotics, prolonged resource exhaustion, growth at high temperatures, and growth within low-nutrient (minimal) media. Surprisingly, there was very little overlap in these adaptive sites, with only 4 out of the 140 appearing under more than one condition. In addition, by comparing these sites with the rest of the protein sequence across bacterial lineages, the authors found that not only adaptation in the laboratory occurs via mutations to highly conserved proteins, but also within the RNAPC proteins, the amino acid sites commonly mutated in laboratory experiments tended to be more highly conserved in nature than other positions within these proteins.

Further analysis identified a number of intriguing patterns. Positions at which adaptation occurred in laboratory experiments also tended to fall within defined protein functional domains, to cluster near each other on the protein structure, and to be located close to the RNAPC active site more often than other sites. To see whether similar dynamics were at play for other proteins, Cohen and Hershberg looked at 19 other proteins containing adaptive mutations associated with resource exhaustion. They found that, as with the RNAPC proteins, sites associated with adaptation in laboratory experiments tended to be more highly conserved among bacteria.

Even more interestingly, when looking at the four selective pressures for which there was sufficient data, these patterns held for antibiotic exposure, minimal media, and prolonged resource exhaustion but not for growth at high temperatures. Thus, adaptations to high temperatures do not exhibit higher conservation, are not clustered near each other or the complex’s active site, and are not enriched within functional domains. As Hershberg notes, it is unclear how common this finding is. “We cannot currently be certain whether adaptations to most conditions behave like the majority of characterized adaptations, with high temperature being an outlier, or whether there are many conditions without data currently available that more closely resemble what is seen for high temperature.”

What is clear is that the dynamics of laboratory adaptation differ greatly from those of natural adaptation. This is because, as the authors explain, “in lab experiments, bacteria are generally exposed to relatively simple, strong, and constant selective pressures. The selective pressures faced within more natural environments are likely far more complex, with several different factors exerting contradictory pressures simultaneously and/or with selective pressures that change with time. Adaptations of the kind that arise so easily during lab evolution may not be so easily permitted within natural environments…Additionally, if such adaptations do occur in response to a specific set of conditions, they may prove to be highly transient, rapidly decreasing in frequency once conditions change.”

In order to explore these questions further, Hershberg believes that it will be “important to try and figure out what these adaptations do in the context in which they are adaptive and to measure their fitness effects under various conditions…Focusing on RNAPC enzyme adaptations could be a useful place to start.” Importantly, such studies could provide new insight into the mechanisms by which evolution occurs, both in the laboratory and in nature. According to Hershberg, “Understanding the reasons for these differences may enable us to learn important lessons on natural adaptation.”
